# Outbreak of caterpillars (Lepidoptera) in Valle de Caracas, Venezuela

**DOI:** 10.1038/s41598-025-21300-2

**Published:** 2025-10-27

**Authors:** David A. Forero-Peña, José A. Suárez, Fhabián S. Carrión-Nessi, José E. Piñango, Daniela Restuccia, Jorge E. Homsi-Álcívar, Iván Mendoza, Juan D. Ramírez, Laura Naranjo-Lara, Alberto E. Paniz-Mondolfi

**Affiliations:** 1 Biomedical Research and Therapeutic Vaccines Institute, Ciudad Bolívar, Venezuela; 2 “Luis Razetti” School of Medicine, Universidad Central de Venezuela, Caracas, Venezuela; 3 Department of Infectious Diseases, Hospital Universitario de Caracas, Caracas, Venezuela; 4 National Research System, National Secretariat of Science, Technology and Innovation, Clayton Panama, Panama; 5 Infecto-Trópico, Panama City, Panama; 6 Africam Safari, Puebla, México; 7 Department of Cardiology, Instituto de Medicina Tropical “Dr. Félix Pifano”, Universidad Central de Venezuela, Caracas, Venezuela; 8 Department of Pathology, Molecular and Cell-Based Medicine, Icahn School of Medicine at Mount Sinai, New York City, NY USA; 9 Infectious Diseases Research Department, Infectus, Bogotá, Colombia

**Keywords:** Lepidopterism, Erucism, Saturniidae, Public health, Entomology, Ecological epidemiology

## Abstract

Lepidopterism, a condition resulting from accidents by caterpillars or the adult forms of moths and butterflies, typically manifests as mild and self-limited hypersensitivity reactions. In August 2023, an unexpected number of accidents by caterpillars were noted in Valle de Caracas, Venezuela. This prospective descriptive study was conducted from August to September 2023. The sample includes both sightings and accidents reported in three regions of Valle de Caracas. A total of 32 sighting reports were recorded, including 13 accidents and 117 caterpillars. The caterpillars primarily belonged to the family Saturniidae, including genus *Dirphia* (86%) (Hübner, 1819) and *Automeris* (9%) (Hübner, 1819). Two caterpillars (2%) were identified as *Megalopyge opercularis* (Smith, 1797). Over half (54%) of the accidents involved children under nine years. Accidents were most common in residential gardens and parks (54%) and public parks and footpaths (31%). Skin lesions were present in all patients, and six patients exhibited systemic symptoms, primarily fever and palpitations. The study highlights a period of increasing accidents by caterpillars in Valle de Caracas, coinciding with multiple sightings of several species of caterpillars, mainly of the genus *Dirphia*. Not only were cutaneous manifestations reported, but cases of lepidopterism were previously unreported for this species in the country.

## Introduction

Out of the more than 165,000 known caterpillar species, only 12 are clinically important, including species of the genera *Automeris* (Hübner, 1819) and *Lonomia* (Walker, 1855) (Saturniidae) and genus *Megalopyge* (Megalopygidae)^1^. The caterpillars can be both poisonous (via hemolymph or other droplets) and venomous (i.e., toxins delivered via setae or spines)^2,3^. These substances are used as a defense system, allowing the caterpillar to respond actively against predators. Recent advances in technology have provided essential information on the composition of venom proteins that has made it possible to relate the effects of the toxins to the symptoms observed in patients^4^. According to their clinical presentation, accidents by caterpillars can be classified into erucism, lepidopterism, dendrolimiasis, ophthalmia nodosa, and consumption coagulopathy with secondary fibrinolysis^5^ (Table 1). However, sometimes these syndromes may overlap^6^.

**Table 1:** Caterpillar envenomation syndromes.

Syndromes	Description
Erucism	Dermatitis caused by contact or airborne exposure to caterpillar irritants
Lepidopterism	Systemic illness caused by direct or indirect contact with caterpillar, cocoon, or moth urticating hairs, spines, or body fluids. Symptoms include upper respiratory and gastrointestinal distress, as well as bronchospasm and dyspnea
Dendrolimiasis	A chronic form of lepidopterism caused by direct contact with caterpillars of the Central Asian pine-tree lappet moth (*Dendrolimus pini*). Symptoms include urticating maculopapular dermatitis, migratory inflammatory polyarthritis, migratory inflammatory polychondritis, chronic osteoarthritis, and rarely, acute scleritis
Ophthalmia nodosa	Chronic ocular condition characterized by initial conjunctivitis, followed by pan-uveitis caused by the penetration of the cornea and migration of irritating hairs into the eye
Consumptive coagulopathy with secondary fibrinolysis	Severe hemorrhagic manifestations. Described in cases of *Lonomia* spp.

Adapted from “Caterpillar Envenomation (Lepidopterism) in a Panamanian Jungle: About a Case”, by Suarez JA, et al., 2023,*Cureus*, 15(12):e51247^5^

Most accidents caused by caterpillars are mild, self-limited hypersensitivity reactions. Episodes are usually isolated and sporadic, and although it is known that temporal, occupational, and geographic factors may influence the risk of developing lepidopterism, its epidemiology is poorly understood. Several clinically important species are subject to dramatic variations in density, resulting in infestations of large numbers of caterpillars or moths and subsequent outbreaks of coincident cases (reproduction synchronization)^7–10^. People working in rural areas (e.g., forestry workers, farmers, entomologists, and gardeners) are at increased risk of exposure, especially during warmer months^7,11,12^. Outbreaks of hypersensitivity reactions to Lepidoptera have also been reported in urban and suburban settings, especially near parks and schools with heavily infested trees^10,13–16^.

In Venezuela, records of accidents by caterpillars are limited to reports and case series. The first cases in the country, which involved fibrinolysis, were published in 1967 in Bolívar state^17^. Subsequently, these caterpillars were identified as larvae of *Lonomia achelous* (Cramer, 1777)^18^. Since then, cases have been reported in Bolívar, Anzoátegui, Apure, Barinas, and Delta Amacuro states (areas near the Orinoco, Caroní, and Apure rivers)^19^. In August 2023, an unexpected number of accidents by two caterpillars (Saturniidae and Megalopygidae families) was observed in three locations of Caracas, Venezuela, with local and systemic manifestations including a small group with cardiovascular alterations mainly related to one of the caterpillars from the family Saturniidae (genus *Dirphia* [Hübner, 1819]), which led to an outbreak investigation. Here we report the findings of that investigation, aiming to characterize the species involved, the clinical manifestations of accidents, and the public health implications of this caterpillar outbreak.

## Results

**Fig. 1:**
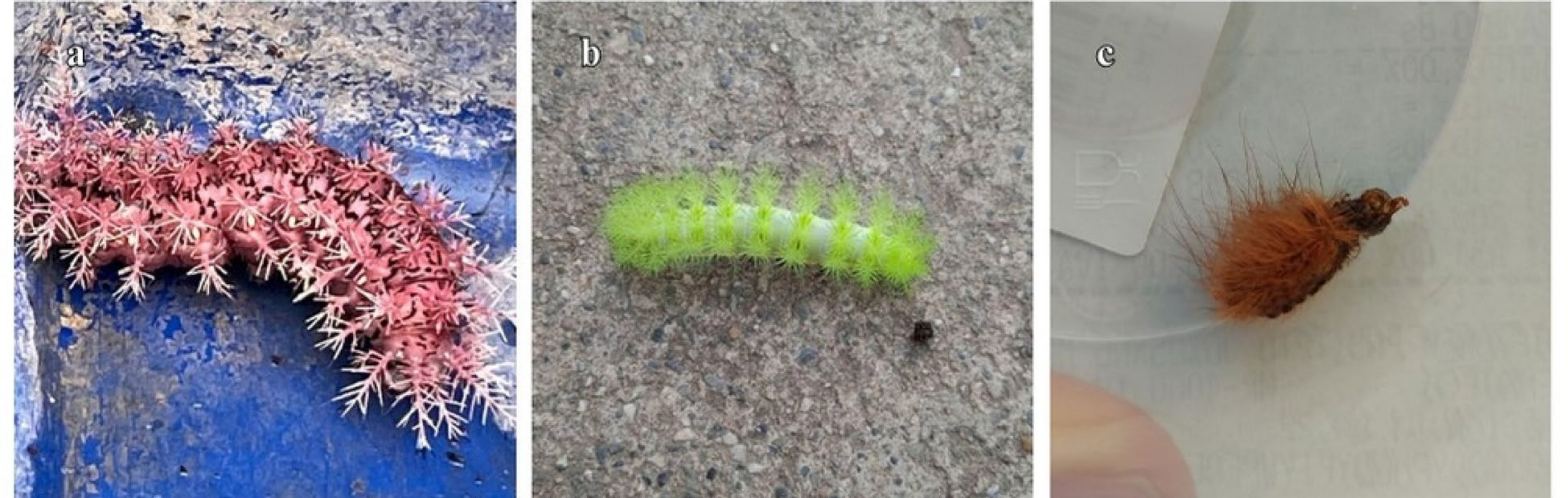
Lepidoptera species found. (**a**) *Dirphia avia* (Stoll, 1780)*.* (**b**) *Automeris* sp. (**c**) *Megalopyge opercularis.*

A total of 32 sighting reports were recorded, including 13 accidents, with 117 caterpillars observed. Most reports occurred in Miranda state, with a total of 115 (98.2%) caterpillar sightings, mainly in Chacao (*n* = 55, 47%), Baruta (*n* = 34, 29%), El Hatillo (*n* = 17, 14.5%), and Sucre (*n* = 9, 7.6%) municipalities. Only two reports (1.7%) were recorded in Capital District. Entomologically, most of the caterpillars were identified as belonging to the family Saturniidae, genus *Dirphia* (*n* = 101, 86.3%), followed by the genus *Automeris* (*n* = 11, 9.4%). Only two (1.7%) caterpillars were identified as *Megalopyge opercularis* (Smith, 1797) (Fig. 1). Most sightings occurred between epidemiological weeks 32 and 34 (August 10–24, 2023), and accidents occurred mainly between weeks 32 and 33 (46.1%).

**Fig. 2:**
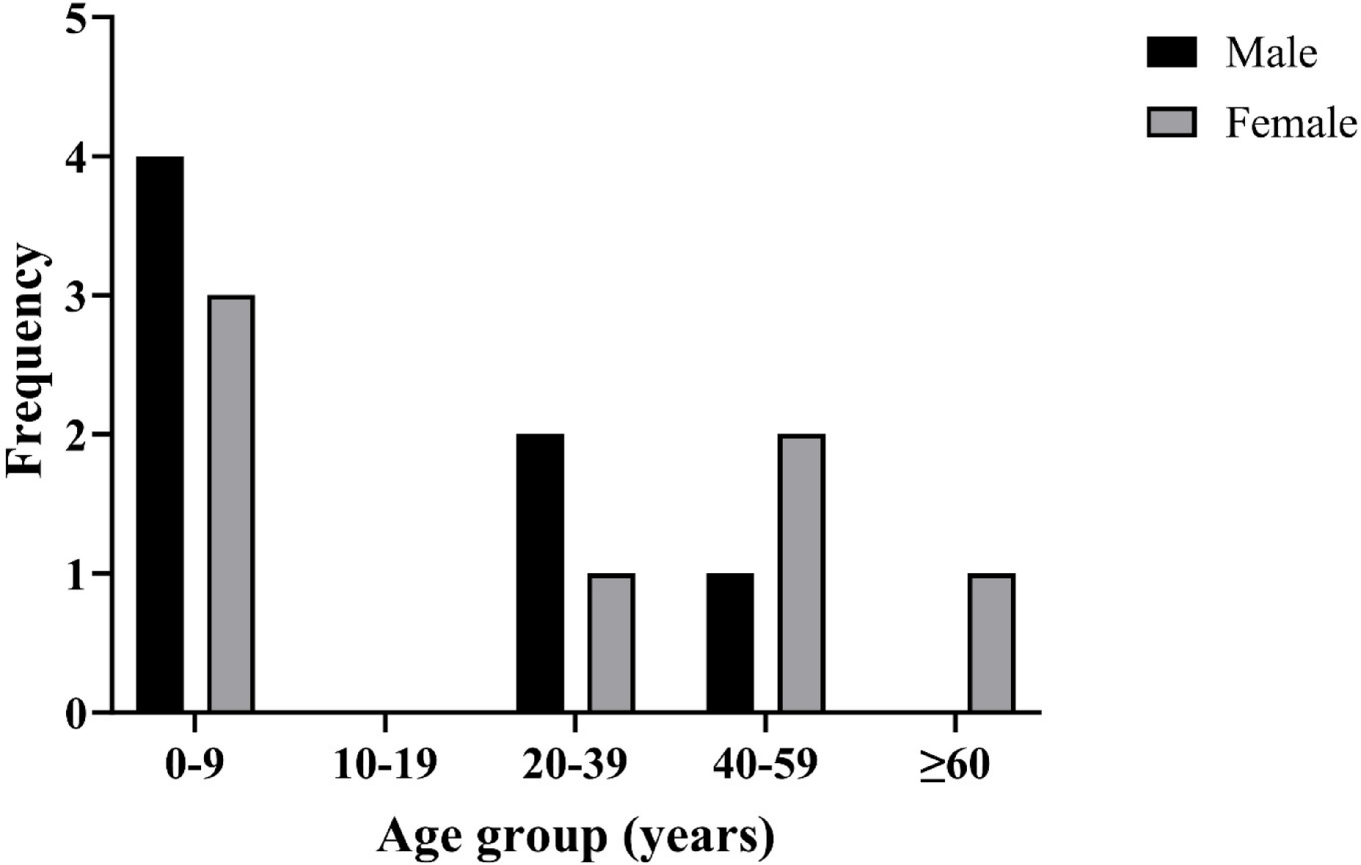
Distribution of the 13 accidents by caterpillar cases by age group and sex.

**Fig. 3:**
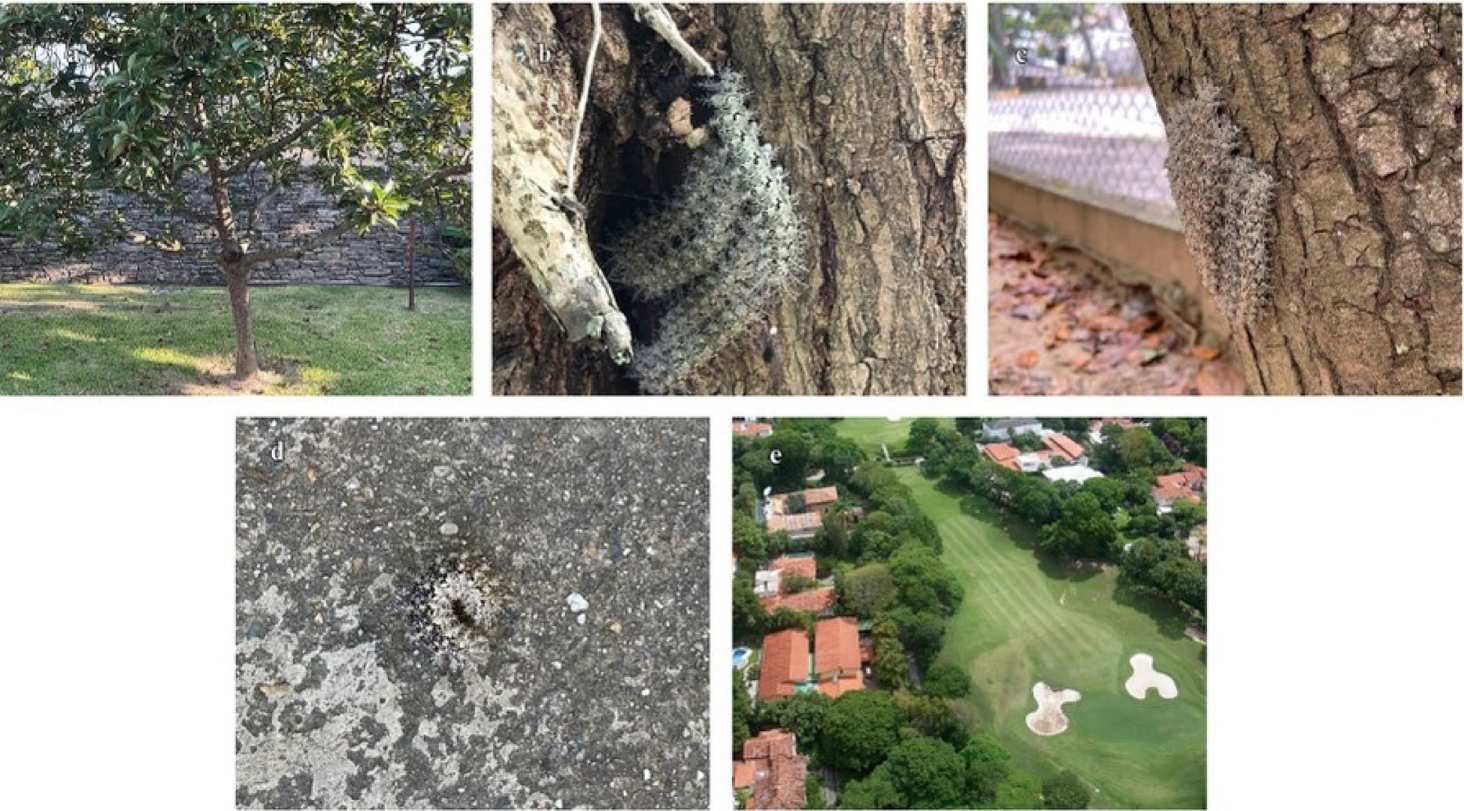
The most frequent locations of accidents by caterpillars reported in the study area. (**a**) Home garden. (**b**) Residential parks. (**c**) Public parks. (**d**) Footpaths. (**e**) Private sports club.

Thirteen accidents by caterpillar contact were documented in seven males (53.8%) and six females (46.2%), with a mean age of 26 years. More than half of the accidents occurred in children under nine years of age (53.8%) (Table 2 and Fig. 2). Chacao was the municipality with the most accident reports (46.1%, *n* = 6), followed by El Hatillo (30%, *n* = 4), and Baruta (23%, *n* = 3). Eleven of the thirteen cases (84.6%) were extra-domiciliary. Most of the reported accidents occurred in residential gardens and parks (53.8%, *n* = 7), followed by public parks and footpaths (30.7%, *n* = 4) and private sports clubs (15.3%, *n* = 2) (Fig. 3).

**Table 2:** Demographic, specimen, and clinical characteristics of 13 patients with accidents by caterpillars.

	Patient 1	Patient 2	Patient 3	Patient 4	Patient 5	Patient 6	Patient 7	Patient 8	Patient 9	Patient 10	Patient 11	Patient 12	Patient 13
*Demographics*													
Age, years	5	3	66	48	51	9	49	9	26	2	5	2	31
Sex	Male	Male	Female	Female	Female	Female	Male	Male	Male	Female	Male	Male	Female
Occupation	Student	Student	Housekeeper	Janitor	Graphic designer	Student	Gardener	Student	Student	N/A	Student	N/A	Merchant
Type of contact	Extradomiciliary	Extradomiciliary	Extradomiciliary	Extradomiciliary	Intradomiciliary	Extradomiciliary	Extradomiciliary	Extradomiciliary	Intradomiciliary	Extradomiciliary	Extradomiciliary	Extradomiciliary	Extradomiciliary
Location of contact	Footpath	Footpath	Public park	Private park	Inside home	Public park	Private park	Inside home	Inside home	Inside home	Social/sports club	Inside home	Social/sports club
*Specimen characteristics*													
Family	Saturniidae	Megalopygidae	Saturniidae	Saturniidae	Saturniidae	Saturniidae	Saturniidae	Saturniidae	Saturniidae	Saturniidae	Saturniidae	Saturniidae	Saturniidae
Genus	*Automeris*	*Megalopyge*	*Automeris*	*Dirphia*	*Dirphia*	*Dirphia*	*Dirphia*	*Dirphia*	*Automeris*	*Automeris*	*Dirphia*	*Automeris*	*Dirphia*
*Clinical characteristics*													
Pain intensity (VAS)	7	6	4	6	8	3	8	10	2	3	5	4	10
Location of lesion	Forearm	Forearm	Thigh	Back	Hand	Forearm	Abdomen	Hand	Hand	Abdomen and leg	Hand	Hand	Hand
Color of lesion	Erythematous	Erythematous	Erythematous	Violaceous	Erythematous	Erythematous	Erythematous with violaceous center	No	Erythematous	Erythematous	Erythematous	Erythematous	Erythematous
Type of lesion	Plaque	Plaque	Plaque	Plaque	Plaque	Plaque	Plaque	Habon	Habon	Habon	Habon	Habon	Plaque
Itching	Yes	Yes	No	Yes	Yes	No	No	No	Yes	Yes	No	No	Yes
Lumpiness	Yes	Yes	No	No	No	Yes	No	Yes	Yes	No	Yes	Yes	No
Other symptoms	No	No	No	Nausea/Vomiting	Myalgias	No	No	Fever	No	No	Fever	Fever	No

N/A: not applicable.

**Fig. 4:**
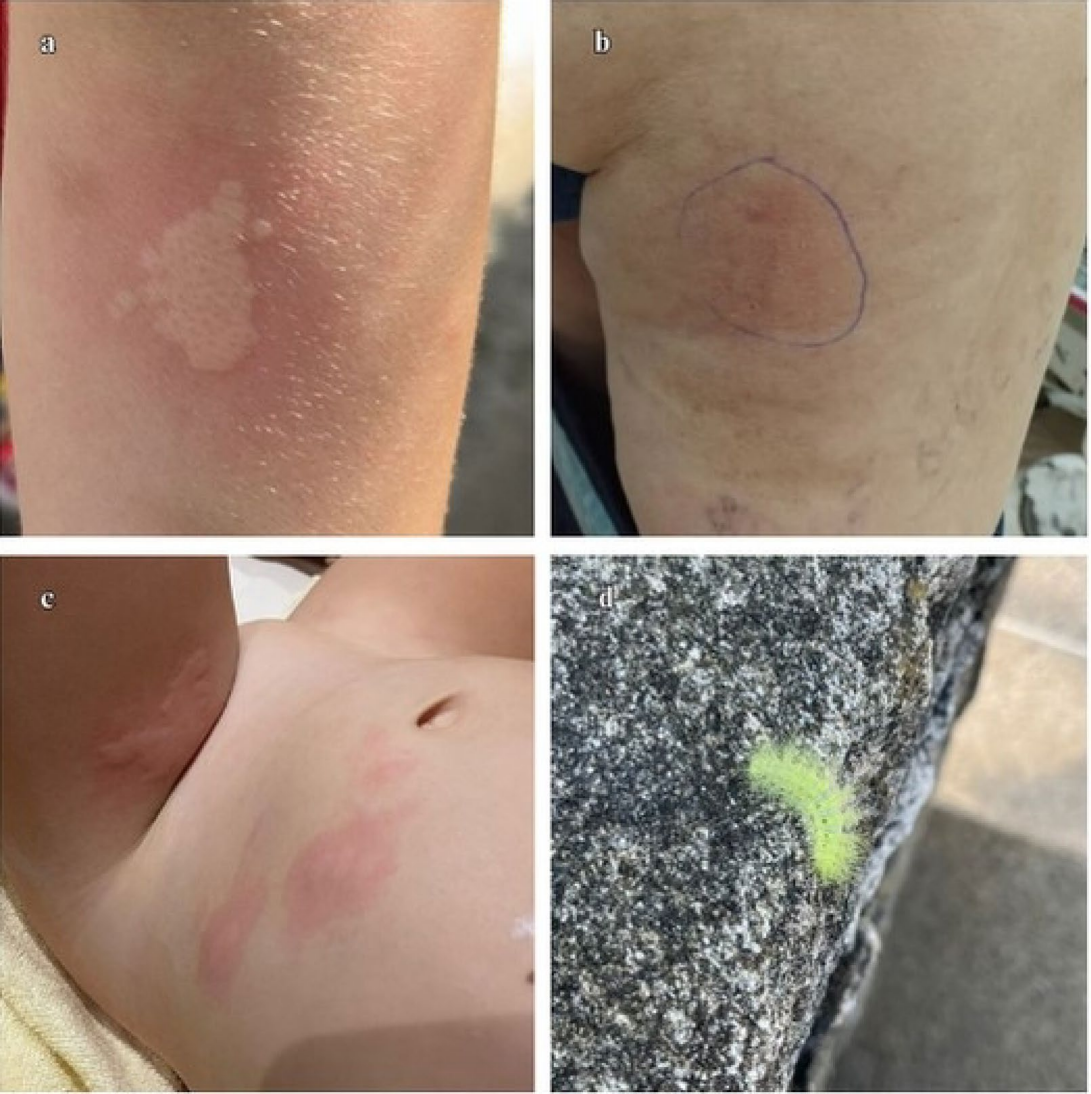
Lesions caused by *Automeris* sp. (Lepidoptera: Saturniidae). (**a**) Erythematous plaque-like lesion in the posterior region of the left forearm, approximately 3 × 2 cm, in a five-year-old boy. (**b**) Erythematous tumefaction type lesion on the second finger of the right anus, approximately 1 cm, in a two-year-old boy. (**c**) Erythematous lesion in the abdomen and anterior face of the left leg, 6 × 6 cm and 5 × 5 cm, respectively, in a two-year-old girl. (**d**) *Automeris* sp. (Lepidoptera: Saturniidae).

**Fig. 5:**
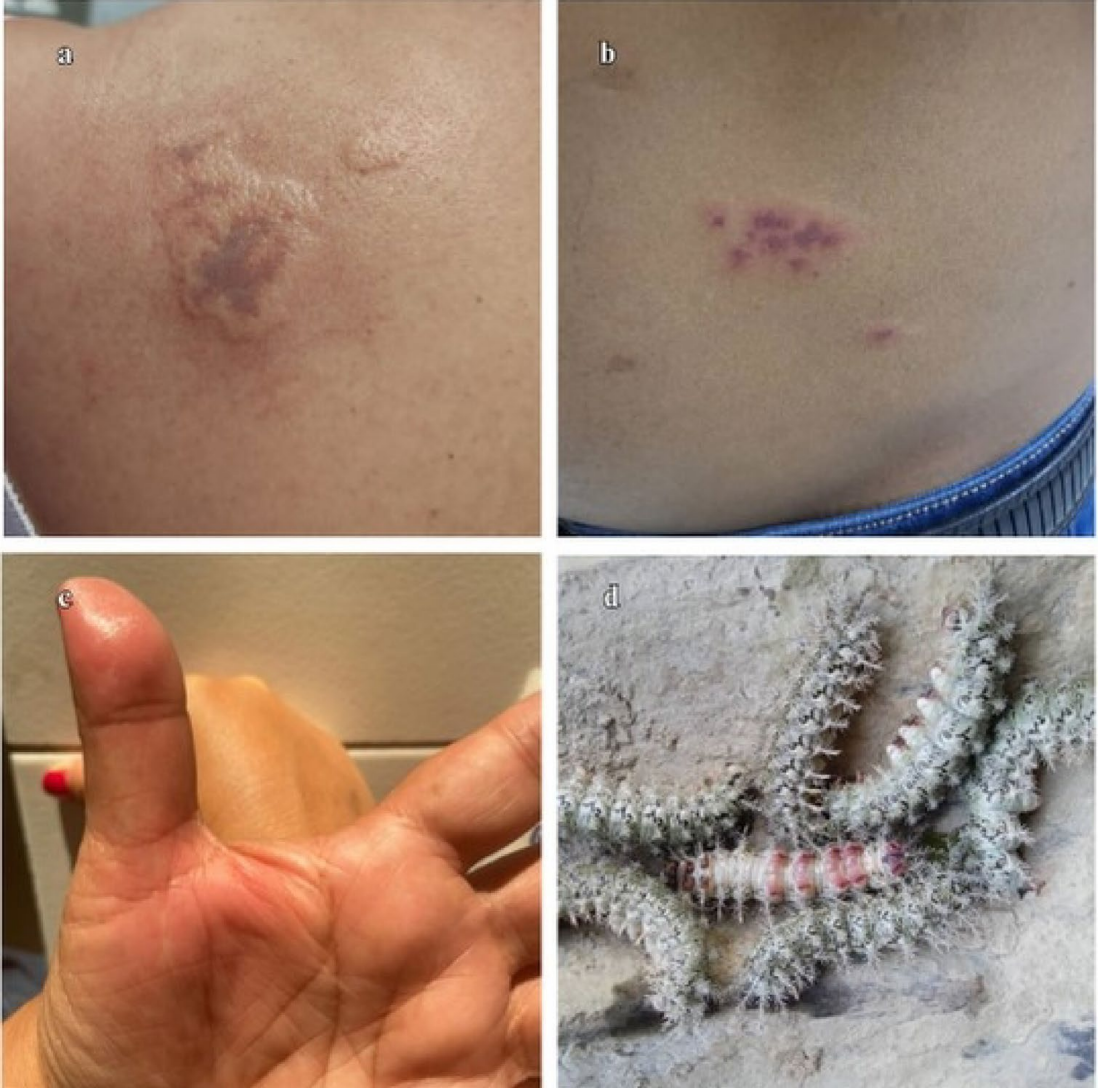
Lesions caused by *Dirphia avia* (Lepidoptera: Saturniidae). (**a**) Violaceous plaque-like lesion in the left scapular region, measuring approximately 6 × 4 cm, in a 48-year-old female. (**b**) Erythematous plaque-like lesion with a violaceous center on the left abdominal flank, measuring approximately 4 × 4 cm, in a 49-year-old male. (**c**) Erythematous plaque-like lesion in the palmar region of the right hand with an increase in volume over its entire length in a nine-year-old male. (**d**) *Dirphia avia* (Lepidoptera: Saturniidae) gregarious.

The most frequent anatomical location of caterpillar contact was the hand (*n* = 5), followed by the forearm (*n* = 3), abdomen (*n* = 2), thigh and leg (*n* = 2), wrist (*n* = 1), and thorax (*n* = 1). Only one two-year-old preschool girl presented multiple locations (thigh and abdomen). All patients manifested burning pain of variable intensity (2–10 on the visual analog scale), with a mean intensity of 6. The most frequent skin lesions were plaques (*n* = 7), more than half of which were pruritic (*n* = 7) (Figs. 4 and 5).

All 13 patients involved in the accidents presented pain at the site of the lesion, where 76.9% described it as burning and 61.5% with an intensity greater than or equal to five on the visual analog scale. Only six patients presented systemic symptoms (45.2%), the most common being fever (*n* = 3), described only in patients under nine years of age, and palpitations (*n* = 3). Other less frequent symptoms (7.6%), such as headache (*n* = 1), irritability (*n* = 1), nausea and/or vomiting (*n* = 1), and myalgias (*n* = 1) were also reported. Each accident was caused by contact with a single caterpillar, except for one patient (case 8) who had contact with seven caterpillars (gregarious) (Fig. 5d).

As for paraclinical examinations, complementary studies were performed as an emergency protocol in only four cases. Among them, a five-year-old boy and a 31-year-old woman presented a prolonged partial thromboplastin time in tests performed within the first 24 h of the accident, a value that normalized after 24 h of the accident in laboratory controls. “Fibrinogen consumption” was documented in 2 patients, a two-year-old boy and a five-year-old boy. Cardiovascular alterations were observed in four patients, including a nine-year-old boy with a negative T-wave from V1–V4 on electrocardiogram, a five-year-old boy with bradycardia and atrial extrasystoles, a two-year-old girl with elevated creatine kinase MB, and a 31-year-old woman with T-wave repolarization disorder. All these alterations were resolved upon follow-up.

## Discussion

To our knowledge, this study represents the first documentation of accidents-by-caterpillar outbreaks in Venezuela. Our research encompasses the recording of sightings and includes accidents involving species from the Saturniidae family (specifically, *Dirphia* sp. and *Automeris* sp.) and Megalopygidae families. Lepidopterism, an allergic reaction triggered by contact with certain caterpillars or adult moth species, has been reported in diverse global locations. For instance, in Eastern Europe, a cohort of 90 individuals, inclusive of 28 children, exhibited clinical symptoms of lepidopterism following both direct and indirect interaction with *Thaumetopoea processionea*^15^. Documented instances of lepidopterism outbreaks associated with the *Orgyia pseudotsugata* species of caterpillars have occurred among boy scouts participating in summer camps in New Mexico^20^. In São Paulo, Brazil, a four-year period saw the report of 568 accidents by caterpillar contact, predominantly among children, with the majority of these incidents attributed to species of the *Megalopyge* genus^21^. In Venezuela, lepidopteran outbreaks have been primarily reported in Monagas, Sucre, and Delta Amacuro states, where instances of lepidopterism caused by both the adult form and the caterpillar of *Hylesia metabus* have been recorded^22^. Most of the literature on accidents by caterpillars caused by lepidopteran species in the region is limited to case reports or small series of cases, mainly involving *Lonomia* in Brazil^23,24^ and Colombia^25^, while we describe here an outbreak that includes 13 accidents by caterpillars mainly caused by *Dirphia* and *Automeris* species, which is less described in the literature^26–28^.

The concurrent sightings and accidents suggest an outbreak. The population dynamics of caterpillars in Valle de Caracas seem to have altered, although the cause of this shift in outbreak periodicity and synchrony remains elusive. Synchrony significantly impacts Lepidoptera population densities, and years with high synchrony can lead to outbreaks. Global climate change leads to a disruption of synchrony between herbivores and their host plants, which may directly impact population viability if natural selection is insufficient to restore synchrony^29^. Moreover, Venezuela’s natural forests are undergoing accelerated deforestation. Over the past five years, the same area has been lost as in the preceding fifteen years, with forest cover diminishing annually^30^. Even in Caracas, rampant deforestation has been condemned^31^, which might alter the caterpillars’ habitat, bringing them closer to communities and reaching epidemic proportions as previously described^14^. Changes in forest ecology, such as tree defoliation^32^ and deforestation^33^, have been linked with clinical outbreaks by caterpillars. Finally, another important factor is the use of insecticides, e.g., in arbovirosis prevention campaigns, both by private individuals and public institutions, which affects both target and non-target insect species^34^. That may have led to a decline in the populations of predators and parasitoids of these lepidopteran species. Collectively, these factors may promote synchronism in Caracas.

Caterpillars from the Saturniidae family are characterized by their sharp, branched spicules, bristles, or hairs. These structures contain a complex blend of bioactive compounds, including enzymes and other toxic components^35,36^. When these substances come into contact with the skin, they trigger a spectrum of dermatological reactions. This syndrome may escalate to multi-organ failure and, in severe cases, death when associated with caterpillars from the *Lonomia* genus^36–41^. Our study predominantly reported the genera *Automeris* and *Dirphia*, which have been previously documented in Panama^42–44^, Colombia^25,45^, and Brazil^45–47^. The Megalopygidae family has also been recorded in Colombia^48^, Argentina^49,50^, Mexico^51,52^, Brazil^53^, Panama^5^, and Venezuela^54,55^. Consistent with our observations, the most frequent clinical manifestations of lepidopteran accidents are erucism and lepidopterism^14,56,57^.

In Venezuela, accidents involving *Lonomia* caterpillars are primarily confined to reports^58^ and case series^18^, predominantly in the southern region. Accidents involving this genus may lead to lonomism, a hemorrhagic diathesis driven by the venom’s procoagulant, fibrinolytic, and phospholipase A2 activity. These results in hypofibrinogenemia, fibrinolysis, decreased coagulation factors XIII and V, plasminogen, and alpha 2 antiplasmin, culminating in a coagulopathy with often severe clinical hemorrhagic manifestations^59^. Although our study did not document any sightings or accidents involving this species, three patients exhibited alterations in partial thromboplastin time levels and fibrinogen consumption, which were normalized within 24 h post-accident, without any hemorrhagic manifestations. These findings are not conclusive to determine whether they were caused by the caterpillars described.

Cardiotoxic effects mediated by cardiomyocyte necrosis have been documented in murine models exposed to *Lonomia obliqua* venom^60^. However, literature on cardiovascular manifestations post-accident is limited, and to our knowledge, no reports exist on cardiovascular involvement related to accidents by non-*Lonomia* caterpillar species. In this study, we report four patients who, following accidents by Saturniidae family caterpillars (genera *Dirphia* and *Automeris*), presented with cardiovascular clinical manifestations, electrocardiographic alterations, and elevated cardiac enzymes. Based on our observations, and given that these cardiovascular findings, we propose a comprehensive cardiovascular evaluation and the necessary ancillary studies to investigate potential cardiac effects from accidents by caterpillars.

Our study is limited to the surveillance of accidents only where the index cases occurred. Although the health system of each of these municipalities was contacted, we received reports of some sightings in other regions, so we cannot conclude that accidents occur exclusively in our study area.

## Methods

### Study site

Caracas is the most urbanized city and the capital of Venezuela. Located in the central-northern coastal region, with an area of approximately 810 km^2^, it has a population of 5,502,992 inhabitants, distributed in five municipalities and 32 parishes^61^. This study focused on Valle de Caracas, located within the Área Metropolitana de Caracas. This valley, which is home to the entire city of Caracas, is crossed by the Guaire River, which has a length of approximately 35 km. Despite its relatively small and irregular size, the city occupies an area of between 80 and 100 km^2^, with a length of 20 km and a width of about 5 km. The city’s meters above sea level (MASL) varies considerably, ranging from 870 to 1043 MASL in the urban area and reaching 900 MASL in its historic center. The highest point in the city is the Naiguatá peak, which rises to 2765 MASL^62^.

### Ethics approval and consent to participate

All procedures involving human participants in this study were approved by the National Centre for Bioethics (CENABI, in Spanish) of Venezuela (CIBI-CENABI-14/2023) and complied with the ethical standards of this committee, as well as the Declaration of Helsinki of 1975, as revised in 2008. All research was performed in accordance with relevant guidelines and regulations. Following an explanation of the study’s nature and survey characteristics, written informed consent was obtained from all participants, or their legal guardians as appropriate. Participants also provided written consent for the use of their medical records for this publication. A copy of each patient’s written consent is available for review by the Chief Editor of this journal.

### Study design and population

A prospective descriptive study was conducted from August 5 to September 7, 2023. The sample includes both sightings and accidents reported in three regions of Valle de Caracas: El Hatillo, Chacao, and Baruta municipalities. Initially, the research team contacted the physicians accountable for the first cases for registration and follow-up. Patients and relatives related to the cases were invited to look for new cases in the area of the accident. For the evaluation of the accidents, the inclusion criterion was to have been in direct contact with one or more caterpillars and to have captured the species for the assessment or to have a photographic record of it. Patients without precise contact exposure or physical or photographic evidence of the caterpillar were excluded.

### Data collection

*Accidents by caterpillars.* For each accident by caterpillars, sociodemographic (sex, age, address), epidemiological (occupation, place of the accident), clinical (local and systemic symptoms), and paraclinical information was collected using the “Google Forms” platform (Google LLC, Mountain View, CA, USA). Subsequently, a face-to-face cardiovascular assessment was performed on patients who attended the call, including anamnesis, physical examination, and electrocardiogram. Finally, patients were classified into one of the following syndromes: erucism, lepidopterism, dendrolimiasis, ophthalmia nodosa, consumption coagulopathy with secondary fibrinolysis, or cutaneous erucism with cardiovascular alterations. For photographic records, each patient was assigned a code that matched the available images of the lesion, the caterpillar, and the accident site if available. For specimen collection, each submitted specimen was assigned a code, linked to the corresponding patient, and preserved in a container with 70% ethanol for subsequent phylogenetic analysis (unpublished data). The identification of each specimen was carried out by an expert observing the photographic images and reviewing the specialized bibliography^63–65^, comparing the diagnostic characteristics of each Lepidoptera family and genus, specifically the presence, arrangement, and shape of the setae or hairs and the coloration of the immature stages. Likewise, comparing these data with the historical records in the associated literature for the affected localities.

*Caterpillar sightings.* A digital announcement was prepared and disseminated with information on accidents and caterpillar sightings, inviting people to report accidents or sightings. Additionally, ambulatory coordinators were contacted and instructed by their healthcare workers to report patients consulting on sightings. A form was created in the “Google Forms” platform for recording sightings and uploading photos of the surroundings, environment, or place where they were sighted as well as of the specimen captured.

### Statistical analyses

Patients’ data were summarized using the following descriptive statistics: mean, standard deviation, median, interquartile range, and/or frequency, percentage. Statistical analyses were performed using SPSS version 26 (IBM Corporation, Armonk, NY, USA). Figure 2 was generated in GraphPad Prism version 10.1.2.

## Conclusions

The study highlights a period of increasing accidents by caterpillars in Valle de Caracas, coinciding with multiple sightings of several species of caterpillars, mainly of the genus *Dirphia*. This described outbreak could result from a synchronicity in the Lepidoptera caused by the different changes and disruptions of the environment, as well as the use of insecticides, which directly impact the population density of these arthropods. Not only were cutaneous manifestations reported, but cases of lepidopterism were previously unreported for this species in the country.

## Data Availability

All data and materials in this article are included in the manuscript.
